# Chlorine dioxide is a broad-spectrum disinfectant against Shiga toxin-producing *Escherichia coli* and *Listeria monocytogenes* in agricultural water

**DOI:** 10.3389/fmicb.2024.1469615

**Published:** 2024-10-25

**Authors:** Jared Van Blair, Alison Lacombe, Beatrice L. Harvey, Vivian C. H. Wu

**Affiliations:** Produce Safety and Microbiology Research Unit, Western Regional Research Center, Agricultural Research Service, United States Department of Agriculture, Albany, CA, United States

**Keywords:** chlorine dioxide, *Escherichia coli*, *Listeria monocytogenes*, water treatment, agricultural water, minimum inhibitory concentration

## Abstract

Agricultural water is commonly treated with chlorine-based disinfectants, which are impacted by water quality. Understanding how water quality influences disinfectants such as chlorine dioxide (ClO_2_) against pathogenic bacteria is important for creating efficacious sanitation regimens. In this study, the minimum inhibitory concentration (MIC) of ClO_2_ needed to achieve a 3-Log reduction against Shiga toxin-producing *Escherichia coli* (STEC) and *Listeria monocytogenes* was compared across agricultural water samples. Sterile ddH_2_O served as a control to compare with environmental samples from Salinas Valley, CA, and laboratory standards. To test different dosages and water qualities, stock ClO_2_ was diluted in 24-well plates with target concentrations of 10, 5, 2.5, and 1.25 mg/L. Well plates were inoculated with pathogens and treated with sanitizer for 5 min. Following treatment, surviving pathogens were enumerated using viable cell counts. The results demonstrate that groundwater samples had the highest water quality of the environmental samples and required the lowest concentration of disinfectant to achieve 3-Log reduction against both bacteria, with MIC between 1.4 and 2.0 mg/L. Open-source samples had lower water quality and required a higher concentration of ClO_2_ for 3-Log reduction, with MIC between 2.8 and 5.8 mg/L for both pathogens. There was no correlation between pH, turbidity, or conductivity/TDS and reduction for either STEC or *L. monocytogenes*, suggesting no individual water metric was driving reduction. A lower dosage was required to achieve 3-Log reduction against STEC, while *L. monocytogenes* required greater concentrations to achieve the same level of reduction. Overall, these results help guide growers in using ClO_2_ as a broad-spectrum disinfectant and demonstrate its efficacy in reaching 3-Log reduction across agricultural water samples.

## 1 Introduction

California’s Salinas Valley is a high-production region for leafy greens (Gorski et al., 2022; Lacombe et al., 2022). Unfortunately, Salinas Valley has been associated with multiple foodborne outbreaks, making it a focal point for investigating produce-related pathogen systems. The Food and Drug Administration (FDA) and the Center for Disease Control (CDC) have stated that agricultural water is a key factor in pathogen conveyance (Gelting et al., 2011; Lacombe et al., 2022). Pathogenic bacteria held commonly in cattle feces are carried by runoff, flooding, and wildlife directly into produce fields or into open-source water, which may be further used in growing operations (FDA, 2021; Gorski et al., 2022; Lacombe et al., 2022). Water treatment is then a critical point to intervene in leafy green production to prevent contamination of food products. Currently, most agricultural water in the United States is treated with chlorine-based sanitizers; however, these sanitizers are labeled to treat fungal pathogens, and no label yet exists for standardizing the treatment of pathogenic bacteria such as Shiga toxin-producing *Escherichia coli*. Federal government agencies, including the FDA and the Environmental Protection Agency (EPA), have more recently developed testing protocols to define standard effective procedures for treating low-quality water (EPA, 2024). This is important because the efficacy of some chemical sanitizers can be heavily impacted by pH and turbidity, while other methods may be more flexible.

Chlorine dioxide (ClO_2_) is an alternative chlorine-based sanitizer used in agriculture water treatment. Aqueous ClO_2_ is a strong oxidizing agent that disrupts bacterial membrane permeability, metabolism, and structural proteins via electrophilic abstraction (Bridges et al., 2020; Jefri et al., 2022; Nadupalli et al., 2011; Ofori et al., 2018, 2017; Zhang et al., 2023). Irrigation infrastructure is vulnerable to the build-up of algal and bacterial growth, and ClO_2_ is useful in biofilm inactivation, making it a diverse tool for growers to sanitize and unclog irrigation lines and other water transport systems (Krüger et al., 2023). Generator-based ClO_2_ requires large infrastructure investment and trained personnel to handle equipment; however, the development of manufacturer kits has made ClO_2_ a simpler and viable alternative for water treatment. This method allows for concentrated batches (200–500 ppm) of aqueous ClO_2_ to be produced with dry precursors. This gives the operator the flexibility to choose the appropriate dose for application based on the manufacturer’s instructions. However, there is a knowledge gap as to the appropriate dose for human pathogen disinfection based on water quality.

Recent Shiga toxin-producing *Escherichia coli* (STEC) outbreaks are a reminder of the importance of reducing contamination in food production operations. The Leafy Greens STEC Action Plan (LGAP) is a document created by the FDA to address repeated leafy-green-related foodborne outbreaks (FDA, 2023). LGAP addresses recurring STEC outbreaks by facilitating collaboration between public and private agency efforts to answer questions regarding produce safety. These efforts serve to reduce the frequency and seriousness of outbreaks (EPA, 2024; FDA, 2021). LGAP included methodology to determine the concentration of chemical sanitizer required to achieve a target of 3-Log (99.9%) pathogen reduction (FDA, 2021). FDA and EPA designed a protocol for testing “worst case scenario” water to test sanitizers’ disinfection efficacy in water with high turbidity, conductivity, total dissolved solids, and varied pH. Due to location and water rights, these water parameters are designed to reflect poor conditions faced by farmers pulling from open-source water systems, including canals and rivers. It is important to define disinfection parameters for these sources so that growers can efficiently inactivate human pathogens before water use.

Another human pathogen, *Listeria monocytogenes*, has been reported across the Salinas Valley in surface waters (Gorski et al., 2022). Long-term sampling and genotyping indicated that the majority of *L. monocytogenes* strains that were isolated contained virulence genes and pathogenicity islands, making them a potential threat to public health and produce safety (FDA, 2023; Gorski et al., 2022). Although *L. monocytogenes* outbreaks are most frequently associated with post-harvest production environments and gaps in good manufacturing practice, the prevalence and pathogenicity of *L. monocytogenes* in Salinas Valley makes it a key organism to monitor in pre-harvest water alongside STEC (Gorski et al., 2022). Therefore, STEC and *Listeria monocytogenes* were chosen for the present study as useful pathogens for testing sanitizing treatments in water samples from California’s central coast growing region.

Following FDA and EPA guidelines, this study aims to define the disinfection efficacy of chlorine dioxide against bacterial pathogens across a wide range of water samples associated with the Salinas Valley, a key production region for leafy greens. Initially, we identified variations in agricultural water samples’ pH, turbidity, conductivity, and total dissolved solids (TDS). Pathogenic bacteria were exposed to a gradient of ClO_2_ dosages to estimate the required concentration of disinfectant to achieve a 3-Log (99.9%) reduction in each water sample. Further, we monitored water metrics at three sites in the Salinas Valley to observe changes in chemistry and microbial content and understand if seasonal changes in water quality exist.

## 2 Materials and methods

### 2.1 Preparation of STEC and Listeria monocytogenes inoculum cultures

Following EPA protocol, cocktails of Shiga-toxin *Escherichia coli* (STEC) and *Listeria monocytogenes* were made to represent pathogens of both gram-positive and gram-negative bacteria. The STEC cocktail included serotypes O157:H7 (ATCC 43888), O121:H19 (BAA-2219), O103:H11 (BAA-2215), O26:H11 (BAA-2196), O111 (BAA-2440), O45:H2 (BAA-2193) and O145:NM (BAA-2192). The *L. monocytogenes* cocktail contained CFSAN 006121, CFSAN002285, CFSAN034257, and CFSAN00078. Prior to the experiment, archived strains of bacteria were pulled from −80°C storage and inoculated into brain heart infusion (BHI) broth. Individual STEC and *L. monocytogenes* serotypes were incubated at 37°C for 24 and 48 h, respectively. Following incubation in BHI, STEC and *L. monocytogenes* were streaked for isolation on Sorbitol MacConkey agar (SMAC) (Neogen, Lansing, MI) and PALCAM agar (Neogen, Lansing, MI), respectively. Colonies were transferred using a 10 μL loop onto Tryptic Soy Agar slants (STEC) and fresh PALCAM plates (*L. monocytogenes*) to be stored at 4°C throughout the experiment. Prior to each experiment, individual serotypes were incubated at 37°C in 7 mL of BHI broth for 24 h (STEC) and 48 h (*L. monocytogenes*). Serotypes of each bacterium were combined into 50 mL conical tubes and centrifuged at room temperature for 5 min at 10,000 × g. Pellets were washed twice using ddH2O prior to the exposure assay.

### 2.2 Preparation of test agricultural water

To represent variable water quality, pH-adjusted double-deionized water (ddH_2_O) (Millipore Sigma, Sigma-Aldrich) was used as a negative control, and samples were taken from a small organic farm (ALBA farms, Salinas Valley, CA), and environmental samples taken from open-source water systems in the Salinas Valley. In addition, two synthetic laboratory standards were made using EPA guidelines at pH levels of 6.5 and 8.4 (EPA_6.5_, and EPA_8.4,_ respectively) (Table 1). The standards were made following the protocol described in the EPA’s agricultural water testing procedure at least 24 h prior to experimentation. Adjustments to pH were made using NaOH and HCl while homogenized with a stir bar. PTI Arizona dust, humic acid, and sodium chloride were used to meet water metric standards, as presented in Table 1.

**TABLE 1:** Guidelines used for the synthesis of the EPA water standards based on the protocol from EPA (2024).

Component	Amount required	Test parameter	Target concentration
Sterile deionized water	1000 ml/L	Total Chlorine	<0.02 mg/L
Arizona test dust	10 mg/L	Turbidity	≥100 FNU
Humic acid	10 mg/L	TOC^1^	>10 mg/L
Table salt	1.6 g/L	TDS^2^	1350–1650 mg/L
HCl and/or NaOH	As needed	pH	6.5 and 8.4

^1^Total organic carbon.

^2^Total dissolved solids. These standards were used to simulate extremely low water quality conditions in sanitizer testing.

### 2.3 Water sample collection

Water samples were collected in the Salinas Valley, CA, during 2023 and 2024. Monthly sampling trips were conducted during June-August and December of 2023 and January and February 2024 to capture seasonal differences. Groundwater from a small organic farm in the Salinas Valley was sampled at two wells that access separate aquifers. The Agricultural Well (AW) assigned for leafy green production was drawn from a well approximately 800 feet deep and was not treated with chlorine. The Domestic Well (DW) samples represented the potable water source and were approximately 500 feet deep. Open-source water sampling sites were identified based on previous research in the area (Gorski et al., 2022) and were located along San Jon Road, Salinas, CA (ENV_1_) and the Salinas River near Gonzales River Road, Gonzales, CA (ENV_2_). These environmental samples represent water systems adjacent to commercial leafy green operations in this region.

### 2.4 Analysis of test water

Each sample was measured for pH, temperature, total dissolved solids, conductivity, free chlorine, and coliforms. The HACH Pocket Pro 2 (Hach Company, Loveland, CO) was used for measuring temperature (°C), pH, conductivity (uS/cm), and total dissolved solids (TDS) (ppm). Turbidity was measured using a HACH 2100Qis Portable Turbidimeter set to measure Formazin Nephelometric Units (FNU) (Hach Company, Loveland, CO). Free and Total Chlorine (F&T) was measured on the HACH Colorimeter DR900 (Hach Company, Loveland, CO) using 10 mL samples supplemented with HACH DPD Free Chlorine PermaChem Reagents (Hach Company, Loveland CO). The presence of coliforms was assessed with 100 mL samples processed through an IDEXX Quanti-tray Sealer Plus (IDEXX, Westbrook, ME) supplemented with Colilert (IDEXX, Westbrook, ME) and reported using the manufacturers Most Probable Number (MPN) table.

### 2.5 Preparation of chlorine dioxide

Stock solution of chlorine dioxide (ICA TriNova, Newnan, GA) was made by submerging a porous sachet containing dry chlorine/salt mixed with acid precursor components in a dark bucket filled with 6 L of ddH_2_O. The aqueous ClO_2_ was allowed to be generated in a chemical hood for 48 h at room temperature until a concentration of ∼250 ppm was achieved. Stock ClO_2_ was then stored at 4°C for the remainder of the experiment. Concentrations were calculated using the DPD method, and color reactions were measured using the Hach DR900 colorimeter. For each reduction assay, the stock concentration was confirmed, and a 10 mg/L sub-stock solution was made using the desired test water in triple-rinsed 250 mL glass bottles and stored at 4°C wrapped in foil prior to assay.

### 2.6 Determination of log-reduction and minimum inhibitory concentration

To estimate the minimum inhibitory concentration (MIC) required to achieve bacterial reduction of 3 log CFU/ml for each sample, a dilution assay was performed utilizing a 24-well plate. The 10 mg/L ClO_2_ sub-stock solution was further diluted across the 24 well plates in test water to final targets of 10, 5, 2.5, and 1.25 mg/L ClO_2_. The negative control wells contained 1 mL of the desired test water with no inoculated culture. To expose the bacterial cocktail to the sanitizer, a 10 uL loop of the cocktail was inoculated into each dilution and timed for 5 min, starting with the first inoculation. To stop the ClO_2_ treatment, the wells were quenched with 1 mL of 1% sodium thiosulfate (Na_2_S_2_O_3_). To enumerate the surviving microbial population, serial dilutions in 0.1% peptone water were performed before plating onto SMAC and PALCAM plates for STEC and *L. monocytogenes*, respectively. Both pathogens were incubated at 37°C, STEC for 24 h, and *L. monocytogenes* for 48 h. All plating was performed using an Eddy Jet 2W Spiral Plater (I&L Biosystems Inc, Königswinterer, Germany). Colony counts were averaged across replicates and transformed into the Log_10_ scale for reporting.

### 2.7 Minimum inhibitory concentration to achieve 3-log reduction

**FIGURE 1:**
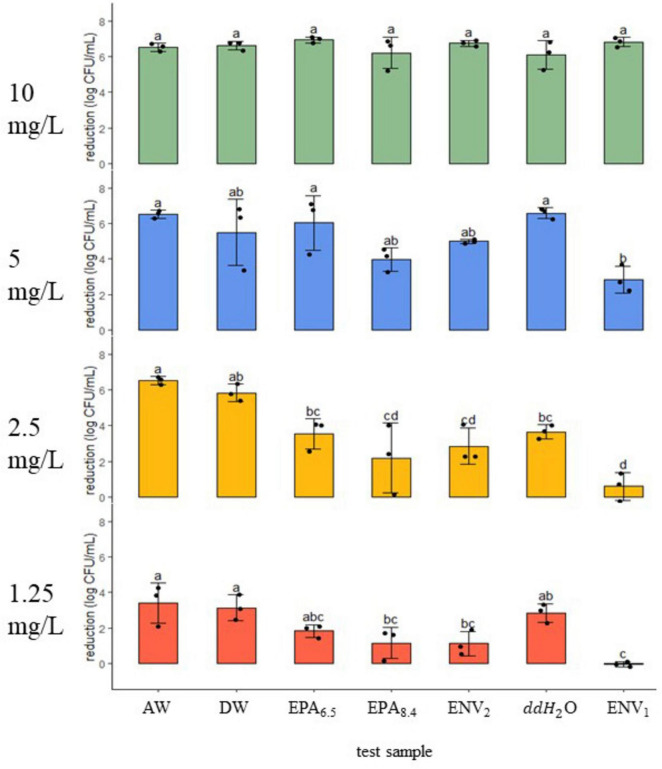
STEC reduction (log CFU/ml ± SD) after a 5-min exposure to a gradient of chlorine dioxide dosages in seven water samples. Dosage (mg/L) is shown on the left of each panel at 10, 5, 2.5, and 1.25. Black dots represent the individual data points. Means (*N* = 3) with similar letter designations represent similar reductions across water samples as determined by two-way ANOVA and Tukey *post hoc* tests (α = 0.05). AW, agricultural well; DW, domestic well; EPA_6.5_/EPA_8.4_, laboratory standards with adjusted pH; ENV_1_/ENV_2_, open source environmental samples; ddH_2_0, double distilled water.

**FIGURE 2:**
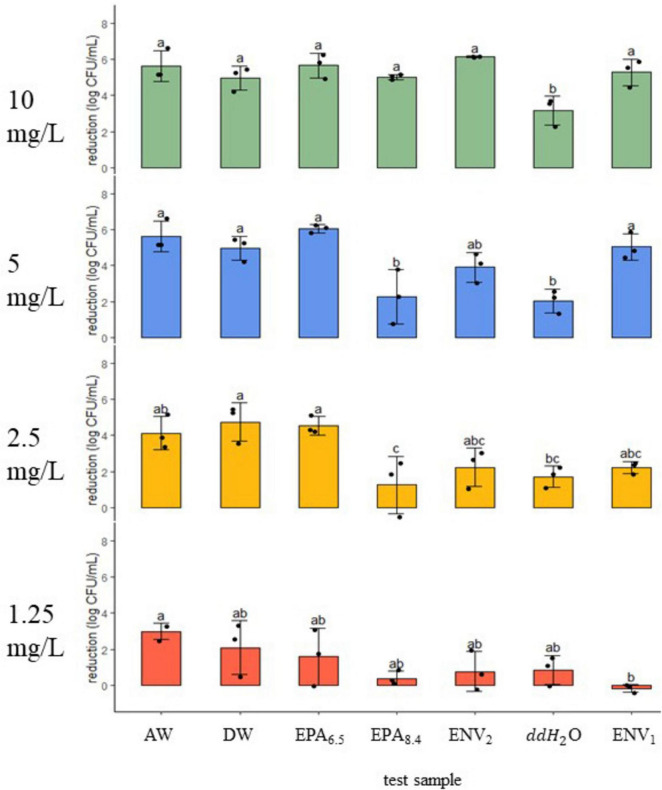
*Listeria monocytogenes* reduction (log CFU/ml ± SD) following 5-min exposure to a gradient of chlorine dioxide dosages in seven water samples. Dosage (mg/L) is shown on the left of each panel at 10, 5, 2.5, and 1.25. Black dots represent individual data points. Means (*N* = 3) with similar letter designations represent similar reductions across water samples as determined by two-way ANOVA and Tukey *post hoc* tests (α = 0.05). AW, agricultural well; DW, domestic well; EPA_6.5_/EPA_8.4_, laboratory standards with adjusted pH; ENV_1_/ENV_2_, open source environmental samples; ddH_2_0, double distilled water.

The lowest concentration of antimicrobials required to achieve a target reduction is the Minimum Inhibitory Concentration (MIC). With a target of 3-Log reduction, MIC was calculated using equations from 2nd-order polynomial lines fitted to reduction data from Figures 1, 2. First and Second-order polynomial fitted lines and equations are shown in Supplementary Figures 1, 2. To estimate the concentration required for 3-Log reductions, equations from each water sample were set to LogN/N_0_ = −3, where the y-axis represents LogN/N_0_, and the x-axis represents the antimicrobial concentration gradient.

### 2.8 Data analysis

All reduction experiments were performed in triplicate (*n* = 3), and all statistical analysis was performed in RStudio (2023.03.1++446) with standard significance levels (α = 0.05). Log reductions were calculated by subtracting treated bacterial populations from respective untreated positive control populations. Two-Way ANOVA followed by Tukey HSD *post hoc* tests were used to determine significant differences in reduction across water samples per treatment group. Student t-tests were used to determine differences in water metrics and sampled bacterial count between seasons. A Spearman’s correlation test was used to assess the strength and direction of the correlation between bacterial reduction and pH, turbidity, conductivity/TDS at 2.5 and 1.25 mg/L treatment levels.

## 3 Results

### 3.1 Test agricultural water quality

All water sample test results reporting pH, free chlorine, turbidity, conductivity, total dissolved solids, coliform, and *Escherichia coli* Most Probable Number are shown in Table 2. Environmental samples ENV_1_, ENV_2_, and the Domestic Well samples had pH of 8.3 ± 0.3, 8.1 ± 0.1, and 8.1 ± 0.2, respectively, which was similar in alkalinity to the EPA_8.4_ control (*n* = 6, *p* < 0.05). However, the Ag Well sample had only slight alkalinity with pH 7.4 ± 0.3 which was between ddH_2_O and EPA_8.4_ (*n* = 6, *p* < 0.05). The ddH_2_O negative control, Ag Well, and Domestic Well samples had no residual chlorine (0 ± 0 mg/L), while EPA_6.5,_ EPA_8.4_, ENV_1_, and ENV_2_ all possessed similar trace levels of residual chlorine (*n* = 6, *p* < 0.05).

**TABLE 2:** Water quality measurements for test agricultural water samples* and controls.

Test water*	pH	Turbidity (FNU)	Free chlorine (mg/L)	Conductivity (uS/cm)	Temperature (°C)	TDS^1^ (ppm)
ddH_2_O	6.7 ± 0.4^C^	0.1 ± 0.05^C^	0 ± 0^B^	2.3 ± 0.6^D^	19.6 ± 1.8^A^	1.9 ± 0.4^D^
EPA_6.5_	6.5 ± 0.01^C^	114.6 ± 0.1^A^	0.4 ± 0.3^A^	1669.3 ± 118.1^A^	18.1 ± 1.4^A^	1283.3 ± 188.3^A^
EPA_8.4_	8.4 ± 0.0^A^	107.6 ± 5.4^A^	0.4 ± 0.3^A^	1797.8 ± 112.4^A^	18.5 ± 0.9^A^	1392.8 ± 164.1^A^
DW	7.4 ± 0.3^B^	1.8 ± 1.1^C^	0 ± 0^B^	719.8 ± 14.6^B^	18.9 ± 3.9^A^	571.8 ± 11.7^B^
AW	8.1 ± 0.2^A^	1.0 ± 0.7^C^	0 ± 0^B^	689 ± 39.1^B^	16.8 ± 1.2^A^	548.5 ± 11.7^B^
ENV_1_	8.3 ± 0.3^A^	72.8 ± 11.6^B^	0.5 ± 0.1^A^	1783.3 ± 34.5^A^	21.6 ± 2.2^A^	1407.5 ± 25^A^
ENV_2_	8.1 ± 0.1^A^	99.7 ± 9.3^A^	0.6 ± 0.1^A^	335.8 ± 69.6^C^	21.3 ± 4.2^A^	267.8 ± 56.3^C^

^1^Total dissolved solids.

*EPA_6.5_, Laboratory standard at pH 6.5; EPA_8.4_, Laboratory standard at pH 8.4; DW, domestic well; AW, agricultural well; ENV_1_, open-source sample 1; ENV_2_, open-source sample 2. Values represent mean (±SD), and significance levels are denoted by letters (*n* = 6).

#### 3.1.1 Turbidity

ENV_2_ had similar turbidity to EPA controls at 99.7 ± 9.3 FNU (*n* = 6, *p* < 0.05). In contrast, ENV_1_, had turbidity measurements of 72.8 ± 11.6 FNU, which was lower than the positive control turbidity (*p* < 0.05). The Ag Well, and Domestic Well samples had low turbidities of 1.0 ± 0.7 and 1.8 ± 1.1 FNU, respectively, similar to the ddH_2_O negative control.

#### 3.1.2 Conductivity and total dissolved solids

There was a significant gradient in conductivity and total dissolved solids (TDS) across water samples (p < 0.001) (Table 2). The ENV_1_ sample had high conductivity and TDS measurements of 1783.3 ± 34.5 uS/cm and 1407.5 ± 25 ppm, respectively, which was similar to the EPA positive controls (*n* = 6, *p* < 0.05). Ag Well and Domestic Well samples had moderate conductivity of 719.8 ± 14.6 and 689 ± 39.1 uS/cm, respectively, which was higher than ddH_2_O (*n* = 6, *p* < 0.05). Similarly moderate, Ag Well and Domestic Well had TDS measurements of 571.8 ± 11.7 and 548.5 ± 11.7 ppm, respectively. The ENV_2_ environmental sample had lower conductivity and TDS measurements of 335.8 ± 69.6 uS/cm and 267.8 ± 56.3 ppm, respectively, significantly lower than the EPA controls but greater than ddH_2_O (*n* = 6, *p* < 0.05) (Table 2).

### 3.2 Response of STEC to chlorine dioxide

Under 10 mg/L treatment, there was 6–7 log reduction in STEC across field and lab samples (*n* = 3, *p* > 0.05) (Figure 1). At the next treatment level of 5 mg/L, the Ag Well, Domestic Well, and ENV_2_ samples showed a similar reduction to ddH_2_O, EPA_6.5_, and EPA_8.4_ controls, with 4–6 log reduction (*n* = 3, *p* > 0.05). However, at 5 mg/L, the ENV_1_ sample had 2.85 ± 0.8 log reduction, which was significantly lower compared to ddH_2_O and EPA_6.5_ controls but not compared to EPA_8.4_ (*n* = 3, *p* < 0.05). When exposed to 2.5 mg/L, Ag Well and Domestic Well groundwater samples demonstrated significantly higher reduction compared to ddH_2_O with 6.51 ± 0.2 and 5.82 ± 0.5 log CFU/ml respectively (*n* = 6, *p* < 0.05). Interestingly at 2.5 mg/L there was no difference in reduction between ddH_2_O, EPA_6.5_, and EPA_8.4_ (*n* = 3, *p* > 0.05). In addition, there was no difference (*p* > 0.05) between ENV_2_ and ddH_2_O control; however, ENV_1_ had a significantly lower reduction than ddH_2_O with only 0.58 ± 0.9 log CFU/ml reduction (*n* = 3, *p* < 0.05). At the lowest treatment level of 1.25 mg/L, the ddH_2_O, EPA_6.5_, and EPA_8.4_ controls had between 1 and 3 log reduction (*n* = 3, *p* > 0.05). Ag Well and Domestic Well samples had slightly higher reductions of 3.38 ± 1.1 and 3.14 ± 0.7 log CFU/ml, respectively, similar to ddH_2_O (*n* = 3, *p* > 0.05). The ENV_2_ sample had 1.10 ± 0.7 log reduction, similar to ddH_2_O, EPA_6.5_ and EPA_8.4_ (*n* = 3, *p* > 0.05). Lastly, the ENV_1_ sample had no reduction, with an average of −0.05 ± 0.2 log CFU/ml.

#### 3.2.1 Minimum inhibitory concentration for a 3-Log reduction against STEC

Minimum Inhibitory Concentration (MIC) for 3-Log reduction against STEC was estimated using equations of lines fitted to reduction data (Table 3 and Supplementary Figure 1). Groundwater samples had the lowest MIC values of 1.4 and 1.6 mg/L for AW and DW, respectively. The ddH_2_O and EPA_6.5_ had similar MIC values of 1.8 and 2.0 mg/L. The open-source sample ENV_2_ MIC was 2.8 mg/L, and the EPA_8.4_ control MIC was 3.7 mg/L. The highest MIC against STEC was 5.8 mg/L for ENV_1_.

**TABLE 3:** Comparing minimum inhibitory concentration (MIC) of aqueous ClO_2_ required to achieve 3-Log reduction against STEC and *L. monocytogenes* across test water samples.

Pathogen	Test water	MIC (mg/L)
STEC	ddH_2_O	1.8
	EPA_6.5_	2.0
	EPA_8.4_	3.7
	DW	1.6
	AW	1.4
	ENV_1_	5.8
	ENV_2_	2.8
*L. monocytogenes*	ddH_2_O	8.3
	EPA_6.5_	2.2
	EPA_8.4_	6.5
	DW	2.0
	AW	1.9
	ENV_1_	3.2
	ENV_2_	3.9

MIC values were calculated using the equation for 2nd-order polynomial models fitted to reduction data (Supplementary Figures 1, 2).

### 3.3 Response of *L. monocytogenes* to chlorine dioxide

At 10 mg/L treatment, the Ag Well, Domestic Well, ENV_1_ and ENV_2_ field samples were similar to EPA_6.5_ and EPA_8.4_ with ∼5–6 log CFU/ml reduction against *Listeria monocytogenes* (*n* = 3, *p* > 0.05) (Figure 2). In contrast, the ddH_2_O negative control had only 3.15 ± 0.8 log CFU/ml reduction, which was significantly lower than the other water samples (*n* = 3, *p* < 0.05). Following 5 mg/L treatment, the Ag Well, Domestic Well, ENV_1_, and EPA_6.5_ control had similar reduction between ∼5–6 log, with ENV_2_ slightly lower at 3.90 ± 0.8 log reduction (*p* > 0.05) (*n* = 3, *p* < 0.05). The ddH_2_O control had significantly less reduction with only 2.03 ± 0.7 log reduction, similar to EPA_8.4_ with 2.26 ± 1.5 log CFU/ml reduction (*n* = 3, *p* > 0.05). At 2.5 mg/L treatment, the Ag Well and Domestic Well samples had 4.12 ± 0.9 log CFU/ml and 4.74 ± 1.1 log CFU/ml reduction, respectively, similar to EPA_6.5_ and higher than ddH_2_O (*n* = 3, *p* < 0.05). The ENV_1_ and ENV_2_ samples had lower reduction values of 2.23 ± 0.3 and 2.23 ± 1.1 log CFU/ml, respectively, which were not significantly different from any controls (*n* = 3, *p* < 0.05). For the lowest treatment of 1.25 mg/L, the Ag Well, Domestic Well, ENV_1_, and ENV_2_ field samples were similar to lab controls with reduction measurements ranging from 0 to 3 Log reduction (*n* = 3, *p* > 0.05). The Ag Well and Domestic Well had the highest reduction at 2.98 ± 0.5 and 2.09 ± 1.5 log CFU/ml while ENV_1_ showed no reduction.

#### 3.3.1 Minimum Inhibitory Concentration for a 3-Log reduction against *L*. *monocytogenes*

Minimum Inhibitory Concentration for 3 Log reduction (MIC) of ClO_2_ against *L. monocytogenes* was estimated using the same method as STEC (Table 3 and Supplementary Figure 2). Groundwater samples AW and DW had the lowest MIC of 1.9 and 2.0 mg/L, respectively. The EPA_6.5_ control and ENV_1_ sample MIC were 2.2 and 3.2 mg/L, respectively. ENV_2_ sample MIC was 3.9 mg/L while EPA_8.4_ control MIC was 6.5 mg/L. The highest MIC against *L. monocytogenes* was in ddH_2_O, at 8.3 mg/L.

### 3.4 Seasonal variation in water metrics

#### 3.4.1 Water metrics

**FIGURE 3:**
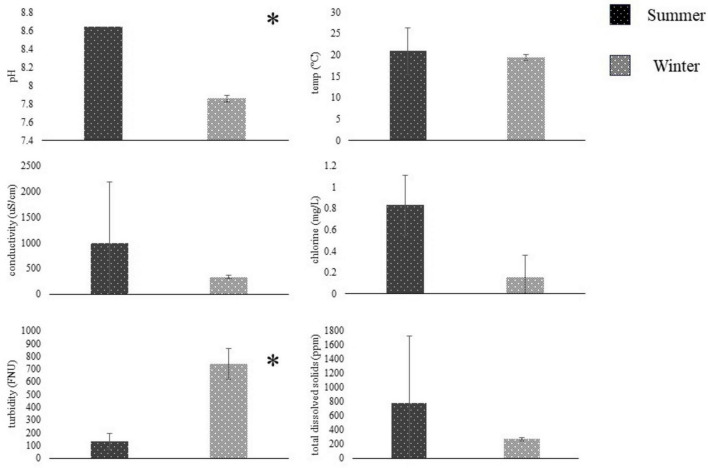
Water metrics (mean ± SD, *N* = 2) compared between summer (dark) and winter (light) seasons using students *t*-test. Summer sampling occurred between June and August, and winter sampling was conducted between November and January. Water was sampled from the Salinas River near Gonzales, CA, and tested on-site for pH, temperature, conductivity, chlorine, turbidity, and total dissolved solids—sampling site based on Gorski et al. (2022). *Represent significant differences (*p* < 0.05) between seasons.

**FIGURE 4:**
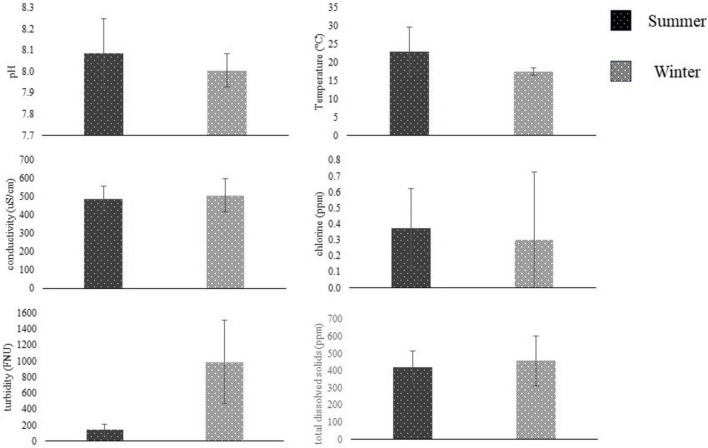
Water metrics (mean ± SD, *N* = 2) compared between summer (dark) and winter (light) seasons using students t-test. Summer sampling occurred between June and August, and winter sampling was conducted between November and January. Water samples were collected from San Jon Rd. canal near Salinas, CA, and tested on-site for pH, temperature, conductivity, chlorine, turbidity, and total dissolved solids. Sampling sites were based on Gorski et al. (2022). *Represent significant differences (*p* < 0.05) between seasons.

Summer and Winter water metrics were compared for each field site in the Salinas Valley using a student *t*-test (*n* = 2). At the ENV_2_ site, there were significant differences between Summer and Winter pH and turbidity (*n* = 2, *p* < 0.05) (Figure 3). At the same time, there were no differences in water metrics between summer and winter samples from the ENV_1_, AW, or DW field sites (*p* > 0.05) (Figure 4). The ENV_2_ site had pH levels of 8.64 ± 0 in the summer and 7.85 ± 0.04 in the winter. The turbidity of ENV_2_ was 131.65 ± 59.89 FNU in the summer and 740.5 ± 122.33 FNU in the winter.

#### 3.4.2 Coliforms and *E*. *coli*

**FIGURE 5:**
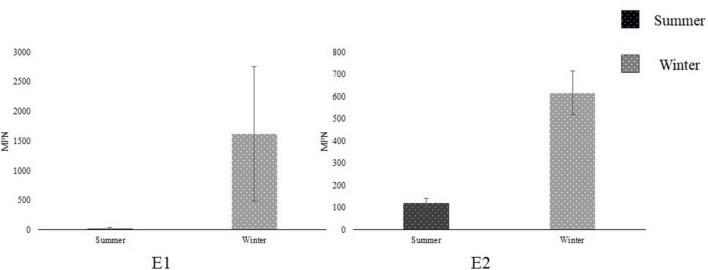
Comparing *Escherichia coli* Most Probable Number (MPN) (mean ± SD) between seasons at open-source sites E1 and E2 in Salinas Valley, CA (*N* = 2) using a student’s *t*-test. Summer (dark) season sampling occurred between June and August. Winter (light) season sampling occurred between November and January. *Represent significant differences (*p* < 0.05) between seasons.

Both open-source water sites tested positive (+) for coliforms in all samples across both seasons. When comparing *E*. *coli* MPN across seasons, there was no significant difference at either the ENV_1_ or ENV_2_ field sites. Both sample sites had a high level of variance from sample to sample, and a general increase in the winter was observed, though not significant (*n* = 2, *p* > 0.05) (Figure 5). The Ag Well and Domestic Well samples tested negative for coliforms and *E. coli* MPN across both seasons, as well as the ddH_2_O control and the EPA control samples.

### 3.5 Water metric and bacterial reduction correlation

Using Spearman correlation, there was no significant correlation between pH, turbidity, or conductivity/TDS and bacterial reduction of STEC or *Listeria monocytogenes* when testing across water samples at 1.25 and 2.5 mg/L (p > 0.05) (Table 4).

**TABLE 4:** Spearman correlation values for assessing the strength of correlation between reduction and water metrics.

Pathogen	Metric vs reduction	P/rho
STEC	pH	*P* = 0.2, *r* = −0.5
STEC	Turbidity	*P* = 0.2, *r* = −0.5
STEC	Conductivity	*P* = 0.9, *r* = −0.07
*L. monocytogenes*	pH	*P* = 0.5, *r* = −0.3
*L. monocytogenes*	Turbidity	*P* = 0.9, *r* = −0.07
*L. monocytogenes*	Conductivity	*P* = 0.2, *r* = −0.5

## 4 Discussion

ClO_2_ is a strong oxidizer and a useful alternative to conventional chlorine-based methods for disinfecting pathogens in various water sources (Barbeau et al., 2005; Bridges et al., 2022; Goodburn and Wallace, 2013). Dry precursor batch treatments of ClO_2_ are efficacious in both ground and open-source waters, suggesting its suitability for agricultural water treatment. The present study demonstrates the inactivation of STEC and *Listeria monocytogenes* using dry precursors to create ClO_2_ in variable agricultural water qualities. It suggests concentrations for a 3-log reduction of human pathogens.

Irrigation water metrics may fall outside optimal NaClO water quality ranges, meriting the need for alternative treatments. Field samples from Salinas Valley had water metrics outside the optimal NaClO treatment range (Table 2). Standard agricultural water treatment utilizes NaClO (bleach) due to low cost and existing infrastructure. NaClO has limitations, including reduced efficacy in water with pH > 8 and reactivity with organic matter, such as nitrogen fertilizers that produce hazardous disinfection byproducts (DBPs) (Chang et al., 2000; Goodburn and Wallace, 2013). In addition, extensive use of NaClO has raised concerns regarding persistent populations of pathogens remaining in the food supply (Hu et al., 2020; Praeger et al., 2018; Xu et al., 2022; Zhang et al., 2023). In this study of ClO_2_ against STEC and *L. monocytogenes*, variation in reduction between water samples appeared at lower dosages while reduction was similar at 5 and 10 mg/L. Across treatments, there was no correlation between sample pH, turbidity, conductivity, and log reduction in either STEC or *L. monocytogenes* (Table 4). While the source of water played a role in water quality and ClO_2_ efficacy, no single variable significantly impacted the inactivation of pathogen. This is in alignment with previous research that demonstrate ClO_2_ broad spectrum ability to inactivate potential pathogens in irrigation water (Truchado et al., 2018).

Groundwater aquifers are a good source of irrigation water due to consistent water metrics and infrequent bacterial contamination. Pathogenic bacteria treated in groundwater required the lowest concentration to achieve 3-Log reduction (Table 3). In addition, Ag Well and Domestic Well samples showed a more significant reduction than ddH_2_O lab water. Recently, Krüger et al. (2023) reported increased ClO_2_ inactivation in phosphate buffered-saline solution compared to tap water when exposing *Pseudomonas aeruginosa, E*. *coli*, and *Staphylococcus aureus* to 0.4 mg/L ClO_2_ for 5 min. Although it is unclear why conductive samples increase ClO_2_ disinfection, a positive correlation between ClO_2_ and factors related to conductivity was demonstrated. Therefore, alkaline groundwater samples possessing moderate conductivity and low turbidity are potential candidates for ClO_2_ treatment.

In contrast to groundwater, open-source water in agricultural areas is subject to frequent contamination by runoff that contributes to fluctuating water metrics and pathogen load (Gorski et al., 2022; Jefri et al., 2022; Lacombe et al., 2022). Open-source field samples ENV_1_ and ENV_2_ had the lowest reduction across water samples despite having more moderate water quality compared to EPA_6.5_/EPA_8.4_ laboratory standards. The complexity and inconsistency in open-source water make it difficult to treat compared to groundwater sources, where high dosage and multiple disinfection methods are introduced to accommodate diversity within the sample. Regional water quality and contamination may vary throughout the year due to seasonal changes in precipitation, temperature, and animal activity. During the 2023–2024 season, extreme variation in water quality throughout seasons was observed at the open-source sites, likely due to sporadic rain events in the 2023–2024 summer-fall-winter. The pH decreased, and turbidity increased significantly at one site (ENV_2_) in the winter (Figure 5). *E. coli* MPN counts were also higher in the winter, coinciding with the elevated turbidity, posing potential issues for water treatment. ClO_2_ is less reactive to organic material and will not break down into chloramines as readily as NaOCl (Jefri et al., 2022; Nadupalli et al., 2011). Several articles report no major influence on ClO_2_ disinfection in pH ranging from 3.0 to 9.0 (Chang et al., 2000; Huang et al., 1997; Jefri et al., 2022; Xu et al., 2022). Therefore, ClO_2_ is well suited for open-source water decontamination because its broader spectrum activities accommodate unpredictable season changes.

Alkalinity is an important factor in determining water quality and the appropriate sanitizer. In the present study, the pH of each sample did not correlate with the reduction in either STEC or *L. monocytogenes* trials, but some patterns were observed (Table 4). Previously, Ofori et al. (2018) demonstrated that ClO_2_ disinfection kinetics increased in alkaline lab waters compared to acidic conditions. Nadupalli et al. (2011) showed that ClO_2_ reactivity increased in hydroxide-rich solutions and stunted reactivity in acidic conditions. While alkaline waters may improve the reactive rates of ClO_2_ oxidation, higher levels of turbidity and organic matter may quickly react ClO_2_ to the less reactive ClO_2_^–^ ion, drastically reducing disinfection (Gagnon et al., 2005; Ayyildiz et al., 2009). In this case, a more significant reduction against STEC in groundwater samples with higher pH (8.1 and 7.4) is expected compared to pure ddH_2_O with a pH of 6.7 (Figures 1, 2 and Table 2). Although pH appears not to be the driving force for variation in reduction, it may play a complex role in influencing ClO_2_.

Bacterial cell envelope structure may influence pathogen tolerance to ClO_2_. In this report, *L. monocytogenes* and STEC responded differently to chlorine dioxide treatment across water samples. Overall, ClO_2_ was less effective in treating *L. monocytogenes* than STEC (Table 3). Previous reports demonstrated that *L. monocytogenes* responded less to ClO_2_ and may be best treated using peroxyacetic acid (PAA) (Hua et al., 2019). Differences in the cell wall and membrane structure between gram-positive and gram-negative bacteria may play a role, as ClO_2_ acts on these features of the cell (Bridges et al., 2020; Hua et al., 2019; Krüger et al., 2023; Ofori et al., 2018). Pathogens also displayed different responses across water samples. For example, *L. monocytogenes* treated in ddH_2_O required the highest dosage to achieve 3-Log reduction compared to any other water sample (Table 3). Little is known about the biotic and abiotic factors that allow *L. monocytogenes* to persist in the environment and whether the lack of environmental stressors may render *L. monocytogenes* harder to inactivate (Gartley et al., 2022; Arcari et al., 2020; Labidi et al., 2023). The EPA updated protocol excluded *L. monocytogenes* from the group of pathogens tested (EPA, 2024). This adjustment reflects the different responses to the environment and disinfectant displayed by *L*. *monocytogenes* compared to STEC, and thus, they should not be treated the same way. Facility contamination remains a pressing concern in food safety, and the abundance of pathogenic *L. monocytogenes* in Salinas Valley waterways underlines the importance of defining effective treatments (Gorski et al., 2022).

Pathogens in this experiment were tested at the stationary phase; however, cells in the long-term survival (LTS) phase exhibit greater tolerance to physical and chemical stressors, including chlorine-based disinfectants (Bhullar et al., 2021; Wen et al., 2009). The long-term survival phase is reached following the death phase and is common for cells persisting in water and soil (Finkel, 2006; Fremaux et al., 2008). While testing disinfectants against cells in the stationary phase is a common practice, understanding how cells in the LTS phase tolerate ClO_2_ treatment in environmental samples will be important for developing treatment recommendations and guidelines.

The primary goal of this report is to inform the development of water treatment guidelines designed to treat human pathogens in agricultural water. In this study, dosages of 1.5–3.5 mg/L ClO_2_ were effective in achieving ≥3 log reduction against stationary phase STEC across water qualities (Table 3). *Listeria monocytogenes* should be treated differently, and higher dosages are required due to the resiliency displayed and the uncertain factors influencing survivability (Table 3). Further mechanism studies may be useful in investigating the process by which gram-positive pathogens react to ClO_2_ and how effective other alternative disinfectants are against *L. monocytogenes* in agricultural water samples.

## Data Availability

The original contributions presented in this study are included in this article/Supplementary material, further inquiries can be directed to the corresponding author.

## References

[B1] Arcari T. Feger M.-L. Guerreiro D. N. Wu J. O’Byrne C. P. (2020). Comparative review of the responses of listeria monocytogenes and *Escherichia coli* to Low pH stress. *Genes* 11:1330. 10.3390/genes11111330 33187233 PMC7698193

[B2] Ayyildiz O. Ileri B. Sanik S. (2009). Impacts of water organic load on chlorine dioxide disinfection efficacy. *J. Hazard. Mater.* 168 1092–1097. 10.1016/j.jhazmat.2009.02.153 19349117

[B3] Barbeau B. Desjardins R. Mysore C. Prévost M. (2005). Impacts of water quality on chlorine and chlorine dioxide efficacy in natural waters. *Water Res.* 39 2024–2033. 10.1016/j.watres.2005.03.025s 15922397

[B4] Bhullar M. S. Shaw A. Mendonca A. Monge A. Nabwire L. Thomas-Popo E. (2021). Toxin–producing *Escherichia coli* in the long-term survival phase exhibit higher chlorine tolerance and less sublethal injury following chlorine treatment of romaine lettuce. *Foodborne Pathog. Dis.* 18 276–282. 33471590 10.1089/fpd.2020.2873

[B5] Bridges D. F. Lacombe A. Wu V. C. H. (2020). Integrity of Escherichia coli O157:H7 Cell Wal and Membranes After Chlorine Dioxide Treatment. *Front. Microbiol*. 11:888. 10.3389/fmicb.2020.00888 32499765 PMC7243733

[B6] Bridges D. F. Lacombe A. Wu V. C. H. (2022). Fundamental differences in inactivation mechanisms of *Escherichia coli* O157:H7 between chlorine dioxide and sodium hypochlorite. *Front. Microbiol.* 13:923964. 10.3389/fmicb.2022.923964 35783445 PMC9247566

[B7] Chang C.-Y. Hsieh Y.-H. Hsu S.-S. Hu P.-Y. Wang K.-H. (2000). The formation of disinfection byproducts in water treated with chlorine dioxide. *J. Hazard. Mater.* 79 89–102. 10.1016/S0304-3894(00)00184-9 11040388

[B8] EPA (2024). *FDA updates protocol for the development and registration of treatments for preharvest agricultural water.* Silver Spring, MD: FDA.

[B9] FDA (2021). *FDA proposes changes to food safety modernization act rule to enhance safety of agricultural water used on produce.* Silver Spring, MD: FDA.

[B10] FDA (2023). *Outbreak investigation of E. coli: Romaine from salinas, California (November 2019).* Silver Spring, MD: FDA.

[B11] Finkel S. E. (2006). Long-term survival during stationary phase: Evolution and the GASP phenotype. *Nat. Rev. Microbiol.* 4 113–120. 16415927 10.1038/nrmicro1340

[B12] Fremaux B. Prigent-Combaret C. Vernozy-Rozand C. (2008). Long-term survival of Shiga toxin-producing *Escherichia coli* in cattle effluents and environment: An updated review. *Vet. Microbiol.* 132 1–18. 18586416 10.1016/j.vetmic.2008.05.015

[B13] Gagnon G. A. Rand J. L. O’Leary K. C. Rygel A. C. Chauret C. Andrews R. C. (2005). Disinfectant efficacy of chlorite and chlorine dioxide in drinking water biofilms. *Water Res.* 39 1809–1817. 10.1016/j.watres.2005.02.004 15899279

[B14] Gartley S. Anderson-Coughlin B. Sharma M. Kniel K. E. (2022). Listeria monocytogenes in irrigation water: An assessment of outbreaks, sources, prevalence, and persistence. *Microorganisms* 10:1319. 10.3390/microorganisms10071319 35889038 PMC9323950

[B15] Gelting R. J. Baloch M. A. Zarate-Bermudez M. A. Selman C. (2011). Irrigation water issues potentially related to the 2006 multistate *E. coli* O157:H7 outbreak associated with spinach. *Agric. Water Manag.* 98 1395–1402. 10.1016/j.agwat.2011.04.004

[B16] Goodburn C. Wallace C. A. (2013). The microbiological efficacy of decontamination methodologies for fresh produce: A review. *Food Control* 32 418–427. 10.1016/j.foodcont.2012.12.012

[B17] Gorski L. Cooley M. B. Oryang D. Carychao D. Nguyen K. Luo Y. (2022). Prevalence and clonal diversity of over 1,200 *Listeria monocytogenes* isolates collected from public access waters near produce production areas on the central california coast during 2011 to 2016. *Appl. Environ. Microbiol.* 88 e357–e322. 10.1128/aem.00357-22 35377164 PMC9040623

[B18] Hu Y. Yang Q. Guo Y. Xu J. Zhou W. Li J. (2020). Volatile organic chloramines formation during ClO2 treatment. *J. Environ. Sci.* 92 256–263. 10.1016/j.jes.2020.02.020 32430128

[B19] Hua Z. Korany A. M. El-Shinawy S. H. Zhu M.-J. (2019). Comparative evaluation of different sanitizers against *Listeria monocytogenes* biofilms on major food-contact surfaces. *Front. Microbiol.* 10:2462. 10.3389/fmicb.2019.02462 31787935 PMC6853887

[B20] Huang J. Wang L. Ren N. Ma F. Juli. (1997). Disinfection effect of chlorine dioxide on bacteria in water. *Water Res.* 31 607–613. 10.1016/S0043-1354(96)00275-8

[B21] Jefri U. H. N. M. Khan A. Lim Y. C. Lee K. S. Liew K. B. Kassab Y. W. (2022). A systematic review on chlorine dioxide as a disinfectant. *J. Med. Life* 15 313–318. 10.25122/jml-2021-0180 35449999 PMC9015185

[B22] Krüger T. I. M. Herzog S. Mellmann A. Kuczius T. (2023). Impact of chlorine dioxide on pathogenic waterborne microorganisms occurring in dental chair units. *Microorganisms* 11:1123. 10.3390/microorganisms11051123 37317097 PMC10224412

[B23] Labidi S. Jánosity A. Yakdhane A. Yakdhane E. Surányi B. Mohácsi-Farkas C. (2023). Effects of pH, sodium chloride, and temperature on the growth of *Listeria monocytogenes* biofilms. *Acta Aliment.* 52 270–280. 10.1556/066.2023.00017

[B24] Lacombe A. Quintela I. A. Liao Y.-T. Wu V. C. H. (2022). Shiga toxin-producing *Escherichia coli* outbreaks in California’s leafy greens production continuum. *Front. Food Sci. Technol.* 2:1068690. 10.3389/frfst.2022.1068690

[B25] Nadupalli S. Koorbanally N. Jonnalagadda S. B. (2011). Chlorine dioxide-facilitated oxidation of the azo dye amaranth. *J. Phys. Chem. A* 115 11682–11688. 10.1021/jp206175s 21923171

[B26] Ofori I. Maddila S. Lin J. Jonnalagadda S. B. (2017). Chlorine dioxide oxidation of *Escherichia coli* in water – A study of the disinfection kinetics and mechanism. *J. Environ. Sci. Health Part A* 52 598–606. 10.1080/10934529.2017.1293993 28301286

[B27] Ofori I. Maddila S. Lin J. Jonnalagadda S. B. (2018). Chlorine dioxide inactivation of *Pseudomonas aeruginosa* and *Staphylococcus aureus* in water: The kinetics and mechanism. *J. Water Process Eng.* 26 46–54. 10.1016/j.jwpe.2018.09.001

[B28] Praeger U. Herppich W. B. Hassenberg K. (2018). Aqueous chlorine dioxide treatment of horticultural produce: Effects on microbial safety and produce quality–A review. *Crit. Rev. Food Sci. Nutr.* 58 318–333. 10.1080/10408398.2016.1169157 27196114

[B29] Truchado P. Gil M. I. Suslow T. Allende A. (2018). Impact of chlorine dioxide disinfection of irrigation water on the epiphytic bacterial community of baby spinach and underlying soil. *PLoS ONE* 13:e0199291. 10.1371/journal.pone.0199291 30020939 PMC6051574

[B30] Wen J. Anantheswaran R. Knabel S. (2009). Changes in barotolerance, thermotolerance, and cellular morphology throughout the life cycle of *Listeria monocytogenes*. *Appl. Environ. Microbiol.* 75 1581–1588. 19168646 10.1128/AEM.01942-08 PMC2655472

[B31] Xu M.-Y. Lin Y.-L. Zhang T.-Y. Hu C.-Y. Tang Y.-L. Deng J. (2022). Chlorine dioxide-based oxidation processes for water purification:A review. *J. Hazard. Mater.* 436:129195. 10.1016/j.jhazmat.2022.129195 35739725

[B32] Zhang Y. Qiu J. Yang K. Lu Y. Xu Z. Yang H. (2023). Generation, mechanisms, kinetics, and effects of gaseous chlorine dioxide in food preservation. *Compr. Rev. Food Sci. Food Saf.* 22 3105–3129. 10.1111/1541-4337.13177 37199492

