# Supplementary material for: Chlorine dioxide is a broad-spectrum disinfectant against Shiga toxin-producing Escherichia coli and Listeria monocytogenes in agricultural water
Source: Front Microbiol. 2024 Oct 25;15:1469615. doi: 10.3389/fmicb.2024.1469615 (PMC11543455; doi:10.3389/fmicb.2024.1469615)
Supplement: Supplementary file 1 [file Data_Sheet_1.docx]

Supplementary Material

**Chlorine dioxide is a broad-spectrum disinfectant against STEC and *Listeria monocytogenes* in agricultural water**

Jared Van Blair*, Alison Lacombe, Beatrice L. Harvey, Vivian C.H. Wu

*** Correspondence:** Vivian C.H. Wu: vivian.wu@usda.gov

# Supplementary Minimum Inhibitory Concentration Material

Polynomial lines fitted to reduction data to estimate minimum inhibitory concentration for 3-Log reduction. The equations from the fitted lines were set to -3 to estimate the concentration of disinfection required to achieve a 99.9% reduction against pathogens. Second-order and first-order polynomial lines were used to model reduction trends with respect to ClO_2_ dosage. Second-order polynomial models were used due to their ability to fit the data trends.

## Supplementary Figures

ddH_2_O

Domestic Well

Ag Well

EPA Standard 6.5

EPA Standard 8.4

ENV_1_

ENV_2_

**Supplementary Figure 1.** First- and Second-order polynomial lines fitted to STEC reduction data were used to estimate a 3-Log reduction per water sample (noted to the left of each graph). The x-axis represents the treatment dosage (mg/L) of ClO_2_ while the y-axis represents Log_10_ reduction ($log\frac{N}{N_{0}}$ ± SD). R^2^ values and equations reported are for second-order polynomials only. The long-dash (purple) line shows the second-order polynomial, while the dotted line represents the first-order polynomial.

ddH_2_O

Domestic Well

Ag Well

EPA Standard 6.5

EPA Standard 8.4

ENV_1_

ENV_2_

**Supplementary Figure 2.** First- and Second-order polynomial lines fitted to Listeria monocytogenes reduction data were used to estimate 3 log reductions per water sample (noted to the left of each graph). The x-axis represents the treatment dosage (mg/L) of ClO_2_ while the y-axis represents Log_10_ reduction ($log\frac{N}{N_{0}}$ ± SD). R^2^ values and equations reported are for second-order polynomials only. The long-dash (purple) line shows the second-order polynomial, while the dotted line represents the first-order polynomial.
